# Characterization and PCR Detection Of Binary, Pir-Like Toxins from *Vibrio parahaemolyticus* Isolates that Cause Acute Hepatopancreatic Necrosis Disease (AHPND) in Shrimp

**DOI:** 10.1371/journal.pone.0126987

**Published:** 2015-05-27

**Authors:** Ratchanok Sirikharin, Suparat Taengchaiyaphum, Piyachat Sanguanrut, Thanh Duong Chi, Rapeepat Mavichak, Porranee Proespraiwong, Bunlung Nuangsaeng, Siripong Thitamadee, Timothy W. Flegel, Kallaya Sritunyalucksana

**Affiliations:** 1 Shrimp-virus interaction laboratory (ASVI), National Center for Genetic Engineering and Biotechnology (BIOTEC), National Science and Technology Development Agency (NSTDA), Yothi office, Rama VI Rd., Bangkok, 10400, Thailand; 2 Center of Excellence for Shrimp Molecular Biology and Biotechnology (Centex Shrimp), Faculty of Science, Mahidol University, Rama VI Rd., Bangkok, 10400, Thailand; 3 Department of Biotechnology, Faculty of Science, Mahidol University, Rama VI Rd., Bangkok, 10400, Thailand; 4 Aquatic Animal Health Research Center, Charoen Pokphand Co. Ltd., Rama 2 Rd., Km 41.5, T. Bangtorat, A. Muang Samutsakorn, Samutsakorn 74000, Thailand; 5 Broodstock Multiplication Center (BMC), Faculty of Marine Technology, Burapha University Chanthaburi Campus, Chanthaburi, 22170, Thailand; Fish Vet Group, THAILAND

## Abstract

Unique isolates of *Vibrio parahaemolyticus* (VPAHPND) have previously been identified as the causative agent of acute hepatopancreatic necrosis disease (AHPND) in shrimp. AHPND is characterized by massive sloughing of tubule epithelial cells of the hepatopancreas (HP), proposed to be induced by soluble toxins released from VP_AHPND_ that colonize the shrimp stomach. Since these toxins (produced in broth culture) have been reported to cause AHPND pathology in reverse gavage bioassays with shrimp, we used ammonium sulfate precipitation to prepare protein fractions from broth cultures of VP_AHPND_ isolates for screening by reverse gavage assays. The dialyzed 60% ammonium sulfate fraction caused high mortality within 24–48 hours post-administration, and histological analysis of the moribund shrimp showed typical massive sloughing of hepatopancreatic tubule epithelial cells characteristic of AHPND. Analysis of the active fraction by SDS-PAGE revealed two major bands at marker levels of approximately 16 kDa (ToxA) and 50 kDa (ToxB). Mass spectrometry analysis followed by MASCOT analysis revealed that both proteins had similarity to hypothetical proteins of *V*. *parahaemolyticus* M0605 (contig034 GenBank accession no. JALL01000066.1) and similarity to known binary insecticidal toxins called '*Photorhabdus* insect related' proteins A and B (Pir-A and Pir-B), respectively, produced by the symbiotic, nematode bacterium *Photorhabdus luminescens*. In *in vivo* tests, it was shown that recombinant ToxA and ToxB were both required in a dose dependent manner to cause AHPND pathology, indicating further similarity to Pir-A and -B. A single-step PCR method was designed for detection of the *ToxA* gene and was validated using 104 bacterial isolates consisting of 51 VPAHPND isolates, 34 non-AHPND VP isolates and 19 other isolates of bacteria commonly found in shrimp ponds (including other species of *Vibrio* and *Photobacterium*). The results showed 100% specificity and sensitivity for detection of VP_AHPND_ isolates in the test set.

## Introduction

Unusual disease outbreaks resulting in severe mortality in cultivated shrimp began in China in 2009 and then spread successively to Vietnam (2010), Malaysia (2011), Thailand (2012) and Mexico (2013) [1–4]. These outbreaks during the early cultivation period of approximately 35 days were characterized by acute hepatopancreatic necrosis in the absence of any accompanying sign of infectious agents in the necrotic tissues. The disease was initially called early mortality syndrome (EMS), but this general designation caused confusion due to other possible causes of early mortality, and a more precise name of acute hepatopancreatic necrosis syndrome (AHPNS) was recommended. When the causal agent was discovered to be unique isolates of *Vibrio parahaemolyticus* from shrimp exhibiting AHPNS in Vietnam, the name of the disease was changed to acute hepatopancreatic necrosis disease (AHPND) [1]. Subsequently, a study in Thailand reported that *V*. *parahaemolyticus* isolates from cultivated Thai shrimp exhibiting histological signs of AHPND were similar to those isolated from Vietnam [4].

AHPND pathology was previously shown by reverse gavage tests to be caused by bacterial toxin(s) present in the cell-free culture broth of VP_AHPND_ isolates and it was suggested that the toxin production might be dependent on plasmid DNA [1]. It was proposed that VP_AHPND_ colonized the shrimp stomach and produced soluble toxins that entered the HP to cause cell sloughing. We hypothesized that the toxins might be proteinaceous and susceptible to fractional ammonium sulfate precipitation from cell-free culture broth of VP_AHPND_ isolates, and that a PCR detection method targeting these toxins might be a convenient tool for identification and detection of VP_AHPND_ isolates. Here we describe the results of testing these hypotheses.

Efforts to control AHPND have been hampered by the lack of a specific and rapid detection method that could be used to determine the reservoirs of the causative bacterial isolates, to insure their absence in shrimp broodstock and post larvae, to monitor shrimp during cultivation and to aid research on possible control measures. Two interim PCR detection methods (AP1 and AP2) were announced on 24 December 2013, updated in 2014 [5] based on purported DNA plasmid sequences present in VP_AHPND_ isolates but not present in non-AHPND *V*. *parahaemolyticus* isolates. Subsequent testing with 80 bacterial isolates at that time revealed that the AP2 method gave superior results to AP1 with 97% positive predictive value for detection of VP_AHPND_ isolates. It was hoped that a method targeting AHPND toxin genes would improve this predictive value. This study aimed to identify and characterize toxins from VP_AHPND_ and to use the toxin information to design a more sensitive and specific PCR method for detection of VP_AHPND_ isolates.

## Materials and Methods

### Experimental shrimp

Since the Ethical Principles and Guidelines for the Use of Animals of the National Research Council of Thailand (1999) apply to vertebrates only and there is no official standard for invertebrates, we adapted its principles to shrimp. We also followed the guidelines of the Australian, New South Wales state government for the humane harvesting of fish and crustaceans (http://www.dpi.nsw.gov.au/agriculture/livestock/animal-welfare/general/fish/shellfish; 30 March 2013) with respect to details regarding the transport of the shrimp and their laboratory maintenance. With respect to processing the shrimp for histological analysis or for killing at the end of an experiment, the salt water/ice slurry method was used as recommended in the Australian guidelines.

Prior to experimental challenges, naïve Pacific whiteleg shrimp *Penaeus vannamei* (2–5g fresh weight) were purchased from local SPF shrimp broodstock producers and acclimated in the laboratory for 2 days in 20 L artificial seawater (Marinium) at 20 ppt salinity with constant aeration in plastic tanks (density 10 shrimp/tank) at ambient temperature (28–30°C). Challenge tests were carried out under the same holding conditions. The shrimp were fed with commercial feed pellets at 5% body weight per day given at approximately 12 hr intervals. Uneaten feed was removed from the tanks 2 hours after each feeding.

### Preparation of bacterial culture broth

Two isolates of VP_AHPND_ (5HP and CN) and one non-AHPND isolate (VPS02) previously described in 2014 [4] were used for comparison of extracellular protein profiles in broth culture. To prepare the culture broth for ammonium sulfate (AS) precipitation, a colony from overnight culture of the appropriate bacterial isolate on tryptic soy agar (TSA) supplemented with 1.5% NaCl was picked and re-suspended in 50 ml fresh culture medium (tryptic soy broth or TSB) supplemented with 1.5% NaCl. The bacterial suspension was incubated at 30°C for 12 hours with continuous shaking. The total 12 hour-culture volume (50 ml) was used as inoculum for 500 ml fresh medium (500 ml) followed by continued cultivation with shaking until the OD_600_ reached 0.6 (approximately 6–8 hours) and was equivalent to approximately 2 x 10^8^ cells per ml. The bacterial cells were removed by centrifugation at 8,500 rpm for 10 min at 4°C. The supernatant culture both was then used to prepare crude protein fractions by ammonium sulfate precipitation.

### Ammonium sulfate precipitation

Ammonium sulphate (AS) precipitation followed a protocol previously described [6]. Briefly, three AS concentrations of 40, 60 and 80% (w/v) were used. After each AS addition, proteins were allowed to precipitate overnight at 4°C before centrifugation at 12,000 rpm for 20 min at 4°C for collection of the pellet and before addition of the next quantity of AS. The AS precipitated fractions were dialyzed against cold-phosphate buffered saline, pH 7.4 (PBS) for at least 3 days with replacement of PBS twice daily. Total protein concentrations were determined using Bradford’s reagent (Bio-Rad) and stored at -80°C.

### Analysis of VP_AHPND_ proteins by SDS-PAGE and mass spectrometry

Protein profiles of the crude protein fractions were analyzed by 10% SDS-PAGE, and protein bands were visualized by staining with Coomassie brilliant blue G-250. Two differentially expressed bands, level with molecular markers of approximately 16 and 50 kDa (called ToxA and ToxB, respectively) were excised from the gel, separately sliced into small pieces (~1 mm^3^) and washed briefly with deionized water before being subjected to alkylation with 50 mM DTT in 50 mM ammonium bicarbonate (AmBIC), pH 8.0 followed by deamidation with 100 mM iodoacetamide in 50 mM AmBIC, pH 8.0. Finally, trypsin digestion was carried out at 37°C for 16 h using 10 ng/ml trypsin (Promega) in 25 mM AmBIC, pH 8.0. After digestion, peptide extraction was carried out using 0.1% trifluoroacetic acid (TFA) in 50 mM AmBic pH 8.0. The solution was reduced in volume by vacuum and then peptides were reconstituted in 0.1% formic acid and analyzed by nano LC-MS/MS (Bruker Daltonics). MASCOT generic files obtained from mass spectrometry were compared with proteins in public databases using the MASCOT database search (http://www.matrixscience.com). The most closely matched proteins were determined based on significant protein hits and *p* < 0.05.

### DNA cloning and sequencing of toxin genes

MASCOT analysis of the mass spectra of ToxA and ToxB gave the highest match for deduced, hypothetical proteins from the genome sequence of *V*. *parahaemolyticus* M0605 (contig034 GenBank accession no. JALL01000066.1) obtained from cultivated shrimp exhibiting AHPND in Mexico [7]. Using the nucleic acid sequences of the matched proteins, PCR primers for *ToxA* and *ToxB* gene targets were designed and used with DNA template from 5HP isolate (VP_AHPND_) for PCR using Phusion DNA polymerase PCR (BioLabs). For *ToxA*, the primers were *ToxA*-Forward: 5ʹ-ATG AGT AAC AAT ATA AAA CAT GAA AC-3ʹ and *ToxA*-Reverse: 5ʹ- GTG GTA ATA GAT TGT ACA GAA-3ʹ. The PCR protocol consisted of pre-denaturation at 94°C for 5 min followed by 30 cycles of 94°C for 30 s, 53°C for 30 s and 72°C for 40 s with a final elongation step at 72°C for 7 min. For *ToxB*, the primers were *ToxB*-Forward: 5ʹ-ATG ACT AAC GAA TAC GTT GTA AC-3ʹ and *ToxB*-Reverse: 5ʹ-CTA CTT TTC TGT ACC AAA TTC ATCG-3ʹ. The PCR protocol was similar to that for *ToxA* amplification except the annealing temperature was 55°C. Amplified PCR products were cloned into pGEM-T EASY Vector (Promega) and transformed into *Escherichia coli* BL21 before being subjected to sequencing. The nucleotide and the deduced amino acid sequences of *ToxA* and *ToxB* were analysed using NCBI-Blast program (http://blast.ncbi.nlm.nih.gov/Blast.cgi) and alignments were performed using Clustal W analysis (http://www.genome.jp/tools/clustalw/).

### PCR detection of ToxA protein gene by the AP3 method

The PCR primers *ToxA*-Forward and *ToxA*-Reverse and the protocol above for amplification of the *ToxA* gene from the VP_AHPND_ isolates 5HP and CN were used in PCR tests with many bacterial isolates to test efficacy of the PCR method in distinguishing between VP_AHPND_ isolates and non-AHPND bacterial isolates commonly retrieved from farmed shrimp. To test the sensitivity and specificity for detection of AHPND isolates (i.e., to test the validity of the detection method), we used 104 bacterial isolates, including 34 non-AHPND VP and 51 VP_AHPND_ isolates (total 85, confirmed by bioassay), plus another 19 isolates of bacteria commonly found in shrimp ponds (see table in results section). The identity of the 85 *V*. *parahaemolyticus* isolates was confirmed by positive PCR results for the lecithin-dependent hemolysin gene (*ldh*) [8]. Bacterial genomic DNA was extracted from 24 hour-broth cultures following the phenol/chloroform method [9]. This PCR method was subsequently named the AP3 detection method for VP_AHPND_. As with the previous AP1 and AP2 detection methods, we do not recommend adapting the AP3 method for nested PCR since this tends to lead to amplification of non-specific amplicons.

Although the 1-step AP3 PCR method was suitable for direct use of DNA extracts from purified bacterial cultures, it was found unsuitable for field samples when levels of AHPND bacteria were low. These samples included shrimp tissues such as those of the HP and stomach, feces from broodstock or juvenile shrimp, whole post larvae, other suspected carriers or tissues thereof and environmental samples such as pond water and sediments. For such samples, we recommend a preliminary enrichment step in any medium suitable for growth of *V*. *parahaemolyticus*. We used TSB containing 1.5% NaCl supplement because it was already being used regularly in our laboratory. Samples should be incubated for 4–6 hr at around 30°C with shaking. After this, any debris is allowed to settle and then the cloudy supernatant is removed and centrifuged to pellet the bacteria. The supernatant solution is discarded and DNA is extracted from the bacterial pellet for use as a template (approximately 100 ng) for each PCR test.

### Heterologous expression of ToxA and ToxB in E. coli

For ToxA expression, the *ToxA* sequence (333 bp) was amplified as described above in a first step prior to using the resulting purified amplicon for a second step PCR with primers containing NdeI and EcoRI restriction sites: *ToxA*-NdeI-Forward: 5’-ACG GCG AGC ATA TGA TGA GTA ACA ATA TAA AAC ATG AAAC-3’ and *ToxA*-EcoRI-Reverse: 5’-ACG GCG GGA ATT CTT AAT GGT GAT GGT GAT GGT GGT GGT AAT AGA TTG TAC AGAA-3’. The PCR product was purified and double digested with NdeI and EcoRI, before ligation into the vector pET-17b (Promega) for transformation of *E*. *coli* BL21 (DE) to produce a fusion protein with a His6 tag. Positive clones were confirmed by PCR using T7 promoter (Promega) and *ToxA*-EcoRI-Reverse primer, and resulting amplicons were purified for further DNA sequencing to confirm in-frame expression. For protein expression, the recombinant plasmid was transformed into *E*.*coli* BL-21 (DE) and then protein expression was induced by 1M IPTG at 37°C for 4 h. The bacterial cells were collected by centrifugation and the recombinant protein was purified using Ni-NTA agarose beads (Qiagen). Purified protein was verified by SDS-PAGE analysis.

For ToxB expression, a similar protocol was followed. The first PCR product was obtained using the PCR method described above and the purified amplicon product used in the second step PCR was carried out using primers containing BamHI and XhoI restriction sites. The primers were *ToxB*-BamHI-Forward: 5’-ACG GCG AGG GAT CCA TGA CTA ACG AAT ACG TTG TAAC-3’ and *ToxB*-XhoI-Reverse: 5’-ACG GCG AGC TCG AGC TAC TTT TCT GTA CCA AAT TCA TCG-3’. The PCR product was purified, double digested with BamHI and XhoI, and ligated into pGEX-4T-1 vector (GE Healthcare) to transform *E*.*coli* BL21 (DE) for expression of a GST fusion protein. Positive clones were checked by PCR and sequenced to confirm in-frame expression. Recombinant protein expression was induced by 1mM IPTG at 30°C for3 h. The bacterial cells were collected by centrifugation and recombinant GST fusion protein was purified by Glutathione Sepharose 4B (GE Healthcare). GST was removed from the purified protein by incubation with 10U/ml thrombin (Sigma) at 37°C overnight. The purified protein was verified by SDS-PAGE analysis.

### Bioassays

By using reverse gavage tests, shrimp were treated with the crude protein fractions prepared by 60% and 80% AS precipitation and then evaluated for induction of AHPND pathology [1]. Before administration, feeding was stopped for at least 6 hours to ensure that the midgut was empty. Ten naïve shrimp each were subjected to reverse gavage using the 60% or 80% AS precipitated fractions (1 μg/ g shrimp). The food grade dye (red, Best Odour Co. Ltd, Thailand) was mixed with the injected material to give visible indication that the preparations had reached the shrimp HP. The control groups were injected with PBS or TSB containing 1.5% NaCl. Cumulative mortality (dead and moribund shrimp) was recorded until 48 hours after injection. Moribund shrimp were fixed with Davidson's fixative [10] and processed for histological confirmation of AHPND pathology. The experiments were repeated twice with two different lots of shrimp and different AS preparations. The same assay protocol was used for testing heterologously expressed ToxA and ToxB proteins. For the control groups, ten shrimp each were injected with BSA at 10 μg/g or 20 μg/g or with ToxA-only or ToxB-only at 5 μg/g or 10 μg/g shrimp. Three combinations of ToxA and ToxB were at 2:2 μg/g, 5:5 μg/g and 10:10 μg/g shrimp were tested. Only the combination of ToxA and ToxB at 10:10 μg/g and BSA control (10 μg/g shrimp) were performed twice, in which the standard deviation (SD) values were calculated, but other combinations and controls were done only once.

### Histological confirmation of AHPND

Slides of shrimp cephalothorax tissue sections fixed and processed for histological examination [10] were viewed using the light microscope for the presence of massive sloughing of HP tubule epithelial cells in the absence of bacteria to confirm AHPND histopathology [1].

## Results and Discussion

### Reverse gavage using the dialyzed 60% AS fraction caused AHPND

**Fig 1:**
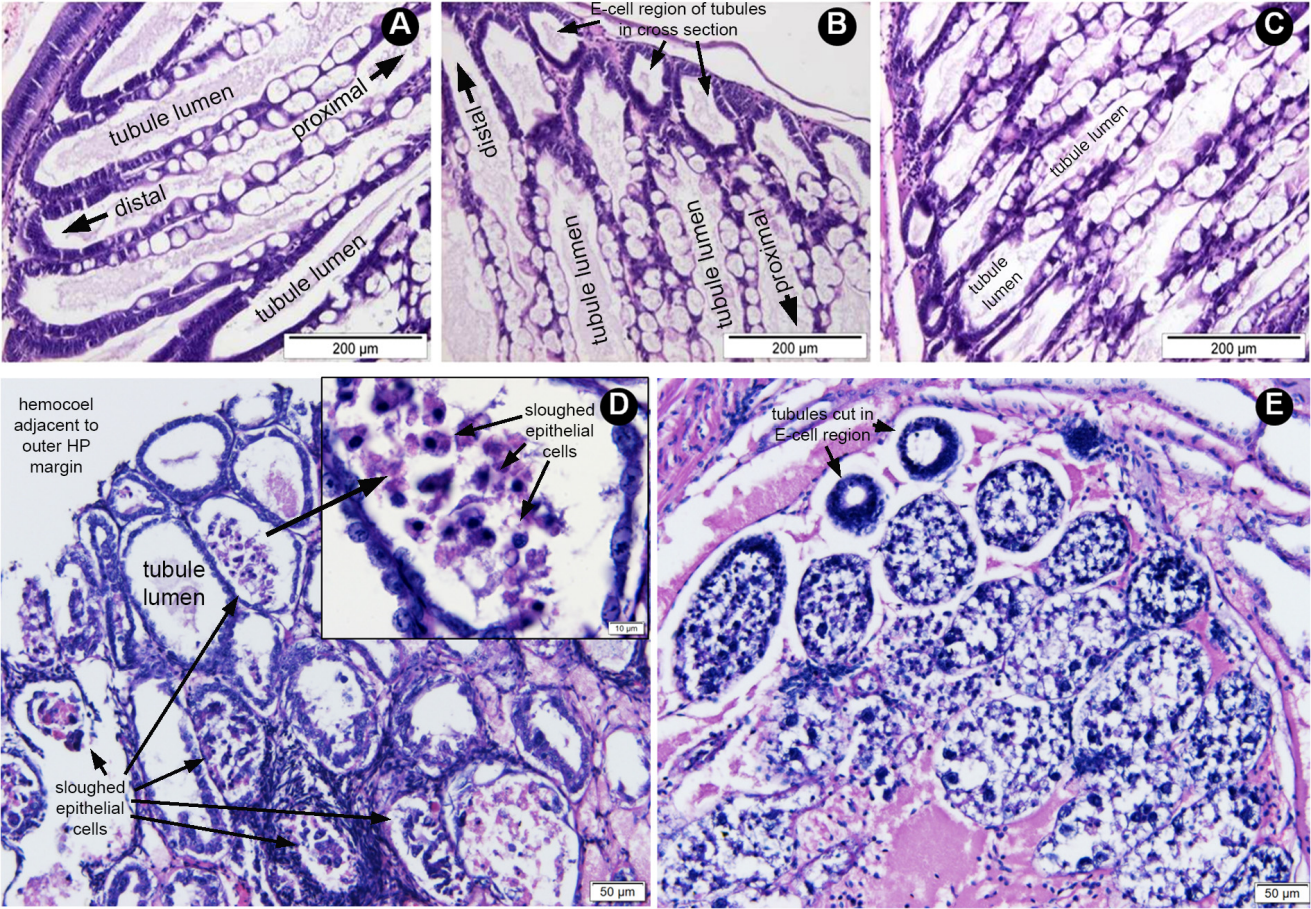
Examples of histopathological sections of hepatopancreatic (HP) tissue from moribund shrimp treated by reverse gavage with 60% AS fractions from *V*. *parahaemolyticus* isolates. (A) A longitudinal section of HP tissue from pre-challenged shrimp showing normal histology with the lumens enclosed by epithelial cell layers comprised of non-vacuolated deeply basophilic (purple stained), embryonic cells (E-cells) at the distal end of the tubule that progress in the proximal direction into a mixture of B-cells with large, single vacuoles, R-cells with multiple vacuoles and F-cells that are non-vacuolated and deeply basophilic. (B) A section of normal HP tissue from the PBS negative control shrimp showing normal tubules mostly in longitudinal section except for a few tubules at the outer (distal) portion of the HP where they are cut in cross-section. The tubule lumens are surrounded by epithelial cells similar to those in (A). (C) Tangential section of HP tissue from shrimp treated with non-AHPND S02 preparation and showing normal HP and showing the same cell types as in (A) and (B). (D) Section HP tubules (mostly in cross-section) from shrimp treated with 5HP preparation and showing AHPND pathology characterized by absence of normal epithelia containing B-cells, R-cells and F-cells as seen in (A) to (C) and instead by massive sloughing of epithelial cells into tubule lumens in the absence of bacteria. The inset shows a magnification of the sloughed epithelial cells in a tubule lumen. (E) Section of HP tubules (cross-section) from shrimp treated with CN preparation and showing AHPND pathology similar to that in (D) but more severe in that all of the tubule lumens are completely filled with sloughed cells except for two tubules cut in cross-section through the E-cell region.

A preliminary study confirmed that shrimp treated by reverse gavage with 12-hour, cell-free, crude culture broth of VP_AHPND_ bacteria exhibited AHPND pathology, as previously reported [1]. We thus hypothesized that if the toxic VP_AHPND_ substances were proteins, they could be fractionally precipitated by stepwise addition of ammonium sulfate (AS) to the cell-free culture broth. The 40%, 60% and 80% AS-precipitate fractions from the VP_AHPND_ isolates 5HP and CN and the non-AHPND VPS02 were subjected to rehydration and dialysis to remove excess AS. Preliminary electrophoresis tests (not shown) revealed that no suspected toxin bands were present in the 40% AS fraction so it was not used in bioassays. When fractions from 60% and 80% were used in reverse gavage bioassays (1 μg total protein each) (**Table 1**), mortality with the 60% AS fraction for 5HP and CN, respectively, was 40% and 70% while there was no shrimp mortality in the shrimp group treated with PBS or with the 60% AS fraction from VPS02. The moribund shrimp showed typical signs of AHPND pathology (i.e., massive sloughing of HP tubule epithelial cells) (**Fig 1**). Mortality also occurred with the 80% AS fractions from 5HP and CN, but histological examination revealed no AHPND pathology in shrimp treated with them (not shown). The latter results indicated that other toxins were present in the broth and that these might exacerbate shrimp mortality while not causing HP cell sloughing. Since there was also high mortality without AHPND pathology in the 80% AS fraction from VPS02, it is possible that some protein(s) present at low levels in the broth were concentrated to a lethal level in the 80% AS precipitate. Differences in protein bands in the 80% AS fractions from the 3 isolates were not studied further because our focus was on the AHPND toxins.

**Table 1:** Results from bioassays of 60% and 80% AS precipitate fractions for shrimp mortality and AHPND histopathology within 48 hours of exposure by reverse gavage (2 replicates of 10 shrimp each).

Crude protein fractions (1 μg/ g shrimp)	Mean percent cumulative mortality± SD	
	60%	80%
PBS (Control)	0	0
S02 (VP)	0	90 ± 0
5HP (VP_AHPND_)	40± 3	44 ± 0
CN (VP_AHPND_)	70± 1	70 ± 0

### SDS-PAGE revealed protein bands unique for VP_AHPND_ isolates

**Fig 2:**
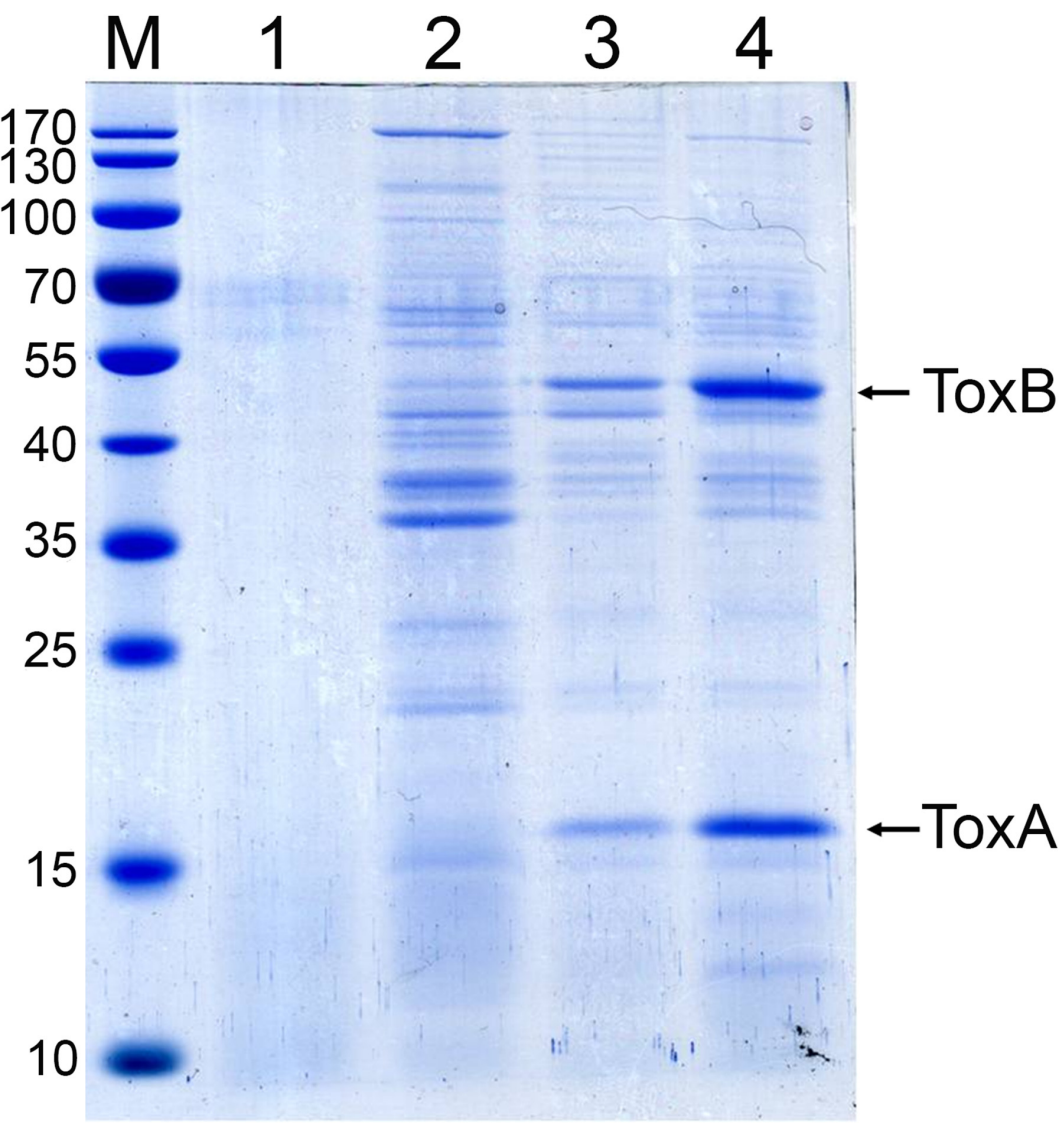
SDS-PAGE analysis of 60% AS fractions from broth of non-AHPND isolate S02 (Lane 2), and VP_AHPND_ isolates 5HP (Lane 3) and CN (Lane 4). Lane 1: 60% AS from culture broth without bacteria. There are 2 major bands (ToxA at ~16kDa and ToxB at ~50 kDa) present in lanes for the VP_**AHPND**_ isolates 5HP and CN, but absent or at low level in non-AHPND isolate S02.

When the 60% AS fractions were analyzed by SDS-PAGE (**Fig 2**), two prominent protein bands with molecular masses (according to the gel marker) of approximately 16 kDa (later named ToxA) and 50 kDa (later named ToxB) were found to be prominent in the fractions from VP_AHPND_ isolates 5HP and CN, but not in the fraction from the non-AHPND isolate VP S02. When these two bands were excised from the gel and subjected to LC-MS/MS analysis, the peptide profiles for the two bands from 5HP matched those from CN. Comparison with known protein records using the MASCOT program revealed that the highest similarities for ToxA and ToxB were for two hypothetical proteins in the draft genome of *V*. *parahaemolyticus* M0605 contig034 (GenBank JALL01000066.1) that originated from AHPND bacteria in Mexico [7]. ToxA matched GenBank protein accession number ETZ12074.1 (MASCOT score 189 with 33% sequence coverage) while ToxB matched GenBank protein accession number ETZ12073 (MASCOT score of 386 with 26% sequence coverage). In this Mexican sequence, it was found that the *ToxA* and *ToxB* genes were closely linked (i.e., separated by 12 nucleotides). From this sequence, primers were designed to amplify the full length *ToxA* and *ToxB* genes from CN and 5HP for cloning and sequencing. The results revealed that the sequences from CN and 5HP were identical to those in JALL01000066.1 and that the two *Tox* genes were also separated by the same 12 nucleotides. After completion of this work, draft sequences of 6 VP_AHPND_ and 4 non-AHPND VP from Thailand were published [11, 12], including the sequence for our 5HP and CN isolates [11]. In all 7 of the VP_AHPND_ isolates listed at GenBank (6 from Thailand and one from Mexico), *ToxA* and *ToxB* were present with identical nucleic acid and deduced amino acid sequences, with identical genome arrangements and separated by 12 identical nucleotides, except that the 5’–3’ orientation of the two protein genes in the Mexican record (JALL01000066.1) was opposite to that in all the other records. Neither of these toxin genes were found in the chromosomal DNA of these AHPND isolates or in the total DNA of the non-AHPND isolates examined [7, 11–13].

Analysis of the full length sequences of *ToxA* and *ToxB* genes showed that they contained 111 and 438 amino acids, respectively. The calculated molecular weights were 12.7 kDa and 50.1 kDa, respectively, with predicted pI of 6.12 and 4.6, respectively. BLASTP analysis revealed 100% homology of ToxA and ToxB proteins to hypothetical proteins AJ90_20630 (accession no. ETZ12074.1) and AJ90_20625 (accession no. ETZ 12073.1), respectively of *V*. *parahaemolyticus* strain M0605, which caused severe mortalities of shrimp in Mexico [7]. ToxA and ToxB also showed significant similarity to known *Photorhabdus/Xenorhabdus* insecticidal related (Pir)-toxins [GenBank CDG18638.1 (33% identity, 50% similarity) and CDG18639.1 (28% identity, 51% similarity), respectively] and to additional hypothetical proteins of *Shewanella violacea* [GenBank WP_013050436.1 (40% identity, 61% similarity) and WP_013050437.1 (44% identity, 63% similarity), respectively].

### Heterologous expression and bioassay of ToxA and ToxB

**Fig 3:**
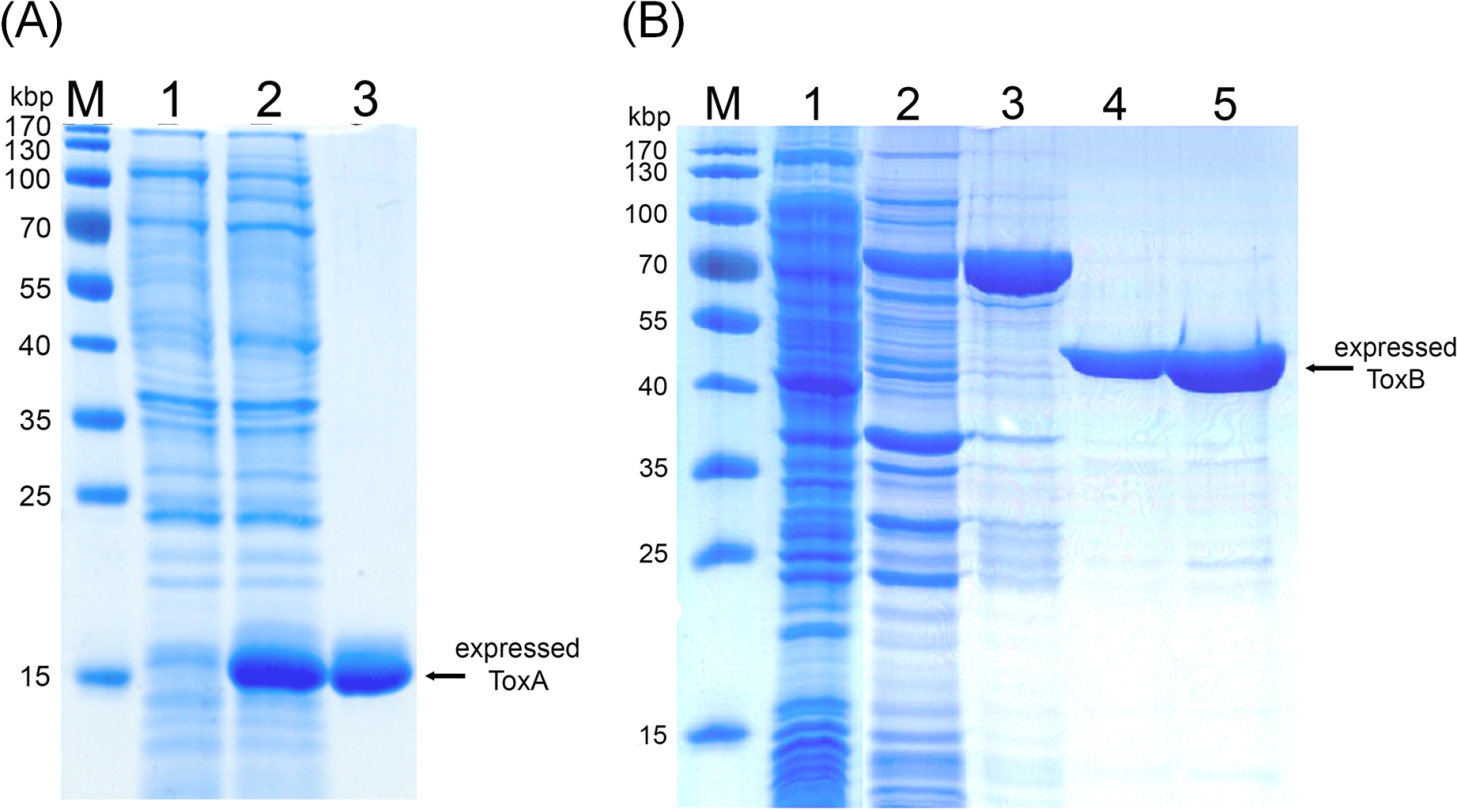
Bacterial expression of ToxA and ToxB. (A) ToxA expressed with a 6-His tag and purified by Ni-NTA affinity chromatography. Lane 1: Bacterial cell lysate from a non-induced bacterial culture; Lane 2: Bacterial cell lysate from an IPTG-induced culture; Lane 3: Eluted protein from the Ni-NTA column. The deduced molecular weight for ToxA-His was 12.7 kDa. (B) ToxB was expressed as a GST-fusion protein. Lane 1: Bacterial cell lysate from a non-induced culture; Lane 2: Bacterial cell lysate from an IPTG-induced culture; Lane 3: Eluted fraction from Sepharose 4B beads; Lanes 4&5: Fraction eluted from Sepahrose 4B after thrombin-cut. The estimated molecular weights for GST-ToxB and ToxB were approximately 76 and 50 kDa, respectively.

**Fig 4:**
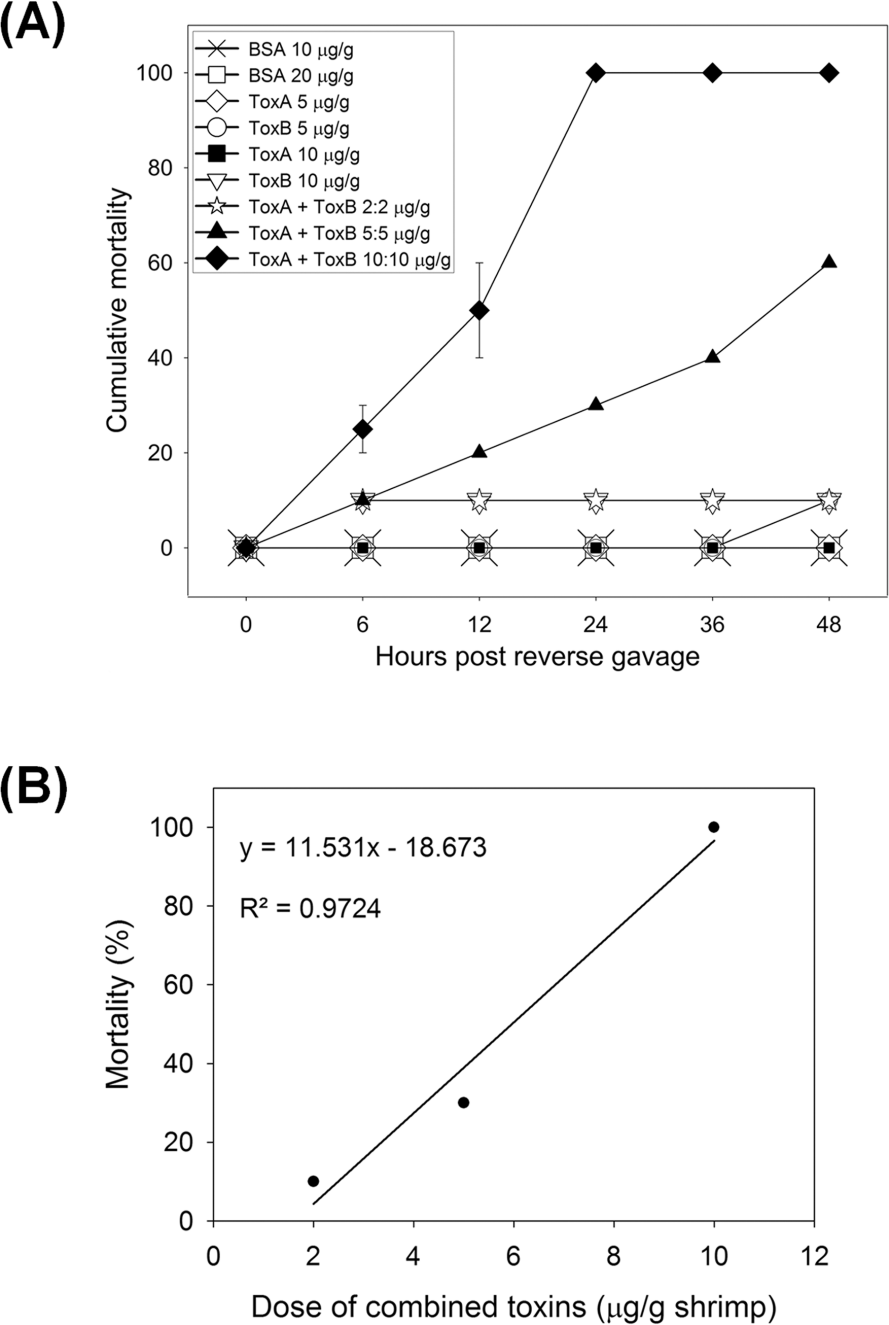
The effect of recombinant ToxA and ToxB administration on shrimp. (A) Graph of cumulative mortality up to 48 h from single and mixed toxins at various concentrations. The graph contains results from two experiments, each with a BSA control (10 ug/g shrimp) (SD = 0) and with co-administration of 10 ug/g each of ToxA+ToxB (SD bars). The other treatments were not duplicated (no SD bars). (B) Graph of mortality at 24 h post administration versus mixed toxin concentration, yielding a linear regression line and rough LD_**50**_ for the mixed toxins of approximately 6 ug/g of each.

**Fig 5:**
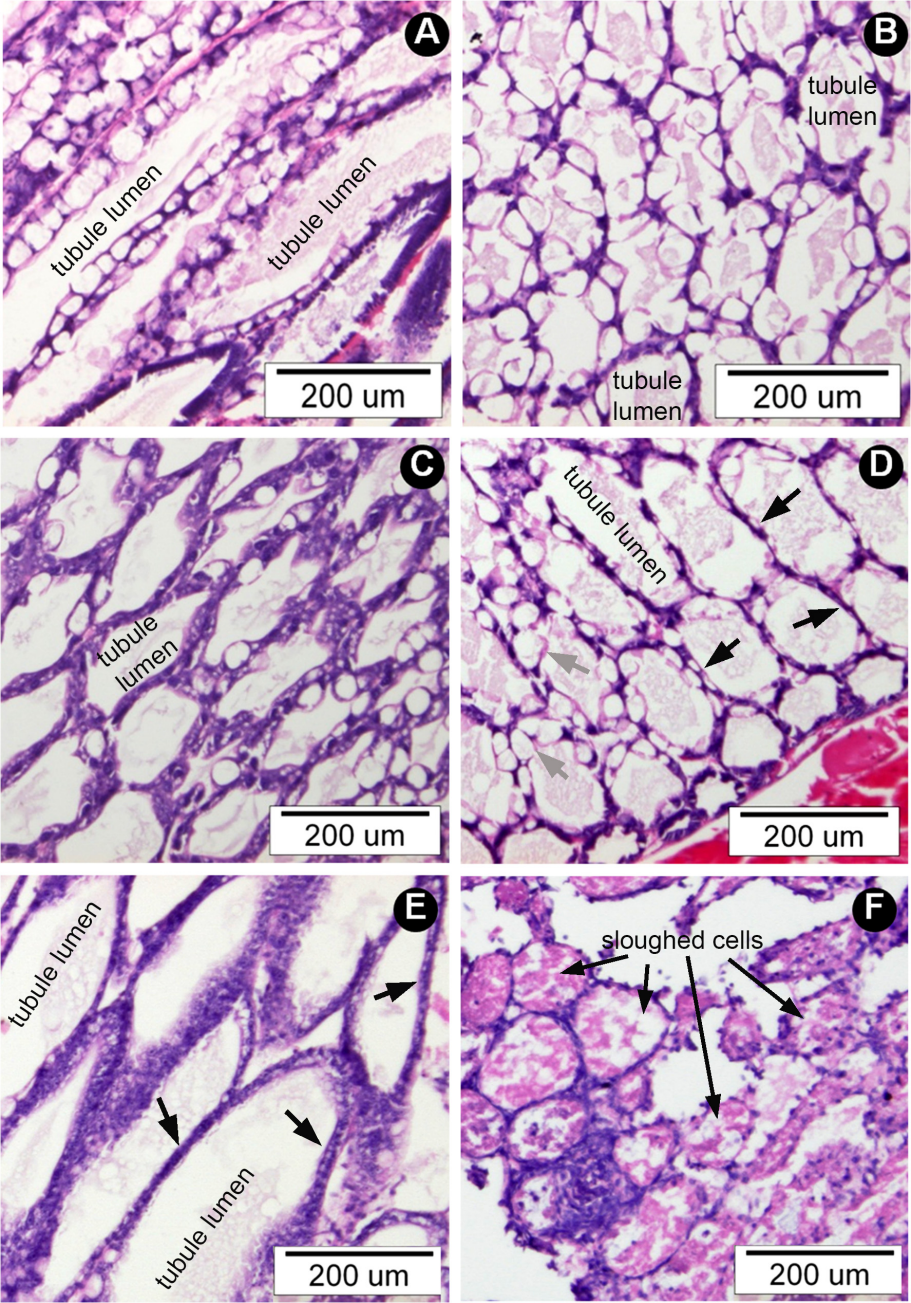
Examples of HP histology of moribund shrimp administered single or combined doses of ToxA and ToxB by reverse gavage. (A) Tissue sections from a BSA (10/20 μg/g) negative control shrimp (tubule longitudinal sections) and (B) a ToxA (5 μg/g) treated shrimp (tubule cross sections) showing only normal histology with morphology and cell types as described in Fig 1A to 1C. (C) ToxB only (5 μg/g) and (D) ToxA+ToxB (2 μg/g each) showing mostly normal histology, but with some thin HP tubule epithelia (black arrows) when compared to epithelia of normal thickness (grey arrows). (E) ToxA+ToxB (5 g/g each) showing enlarged HP tubules (compared to A-C) with collapsed epithelia (black arrows) but no cell sloughing. (F) ToxA+ToxB (10 μg/g each) showing massive sloughing (black arrows) and dissolution of HP tubule epithelial cells (i.e., severe AHPND histopathology).

To test for the ability of ToxA and/or ToxB to cause AHPND, they were heterologously expressed in *E*. *coli*. His-tagged ToxA was purified via Ni-NTA affinity beads (**Fig 3A**) while ToxB fused to GST was treated with thrombin to release it from affinity beads, yielding expressed ToxB (**Fig 3B**). When these two heterologously expressed proteins were tested separately by the reverse gavage bioassay at concentrations of 5 and 10 μg each/g shrimp, it was found that treatment with the BSA negative control at 10 or 20 μg/g or with ToxA at either 5 or 10 μg/g resulted in no shrimp mortality (**Fig 4A**) and was accompanied by normal HP histology (**Fig 5A&5B**) for up to 48 h after administration. By contrast, ToxB at 5μg/g gave 10% mortality (1/10 shrimp) at 48 h while at 10 μg/g it gave 10% mortality (1/10 shrimp) at 6 h after administration, but no more mortality thereafter (**Fig 4A**). In both these ToxB-only treatments, HP histology was normal (similar to **Fig 5A&5B**), including that of the moribund shrimp, except for some thin tubule epithelia in the latter (example ToxB 5 μg/g **Fig 5C**) (i.e., no AHPND pathology), indicating that ToxB alone, even at 10 μg/g could not induce AHPND pathology. The cause of mortality in the 2 moribund shrimp could not be determined.

For treatments of combined ToxA and ToxB at 2 μg/g each, mortality was 10% by 6 h but did not increase thereafter, and HP histology was mostly normal except for some thin tubule epithelia (**Fig 5D**), as with the single ToxB treatments above. At 5 μg/g each, mortality was 60%, but the moribund shrimp did not show typical AHPND pathology. Instead, they showed many enlarged tubules with markedly collapsed epithelia (**Fig 5E**). Only at 10 μg/g each was mortality 100% by 24 h and accompanied by massive sloughing of tubule epithelial cells characteristic of AHPND (**Fig 5F**). Using the data for three doses of the mixed toxins at 24 h, a rough 24 h-LD_50_ for the two-toxin mixture was found to be approximately 6 μg each/g shrimp (**Fig 4B**).

In summary, these toxicity assays revealed that neither of the heterologously expressed ToxA or ToxB proteins alone could induce typical AHPND pathology and that a combined dose of 10 μg/g each was required to do so. The low mortality (1/10 shrimp) from ToxB alone at 5 or 10 μg/g may or may not have been caused by ToxB. Although it might be argued that more rapid death by 6 h for the 1 shrimp at 10 μg/g compared to 48 h for the 1 at 5g/g indicated a dose relationship, but the lack of further death after the first 6 hours with 10 μg/g weighs against that argument. On the other hand, the marked thinning of the HP tubule epithelium in the mixed toxin dose of 5μg/g each is similar to the results previously obtained when concentrations of VP_AHPND_ isolates were lowered [4], supporting an earlier suggestion that some isolates of AHPND bacteria with reduced virulence may cause mortality accompanied by HP tubules that showed collapsed epithelia instead of cell sloughing. Despite these qualifications, the results clearly indicated that both ToxA and ToxB together were necessary to cause typical AHPND histopathology.

Curiously, the quantity of combined ToxA and ToxB proteins required to cause 100% mortality accompanied by AHPND histopathology was 10 μg/g shrimp each while the quantity of the crude 60% AS precipitate needed to achieve the same result was only 1 μg total protein/g shrimp. No tests were carried out to determine the reason for this discrepancy, but possibilities include the facts that the expressed ToxA protein we employed still had an artificial 6His tag, that the ratio of the two toxins may have been non-optimal and that other synergistic toxins may have been present in the crude 60% AS precipitate. In favor of the latter possibility is the fact that the 80% AS fraction caused high shrimp mortality but no AHPND pathology, indicating that it contained other toxins. Thus, other components of the 60% AS precipitate may act in a synergistic or additive manner to increase the potency of the ToxA-ToxB combination. More work is needed to clarify these phenomena.

The requirement for both ToxA and ToxB together to cause AHPND pathology supported the sequence homology search results revealing that the AHPND ToxA and ToxB most closely resembled the Pir-A and-B insecticidal toxins from the bacterial genera *Xenorhabdus* and *Photorhabdus* [14]. These gram-negative and nematode-symbiotic proteobacteria are in the family *Enterobacteriaceae*, and *Photorhabdus* species colonize the intestines of entomopathogenic nematodes from the genus *Heterorhabditis* [15]. *Heterorhabditis* attacks insects at the larval stage and releases *Photorhabdus* from their intestines into the insect hemocoel where they proliferate rapidly and secret toxins that kill insect hosts. Like ToxA and ToxB here, the Pir toxins of *P*. *luminescens* are located at two distinct loci in the *P*. *luminescens* TT01 genome and injection of either Pir-A or Pir-B alone does not kill insect hosts [16] while combined injection does [17]. Despite this additional similarity, the sequences of the Pir-A&-B toxins do not have a high protein sequence identity with respective ToxA and ToxB from VP_AHPND_ isolates, so the mechanism of action for the insect and shrimp toxins may not be identical. Less is known about the two hypothetical proteins of *Shewanella violacea* that also show sequence homology to our ToxA and ToxB, but they too may be worthy of further examination, since some *Shewanella* species are also known to be pathogens of aquatic animals [18].

### Successful PCR detection of VP_AHPND_ isolates

**Fig 6:**
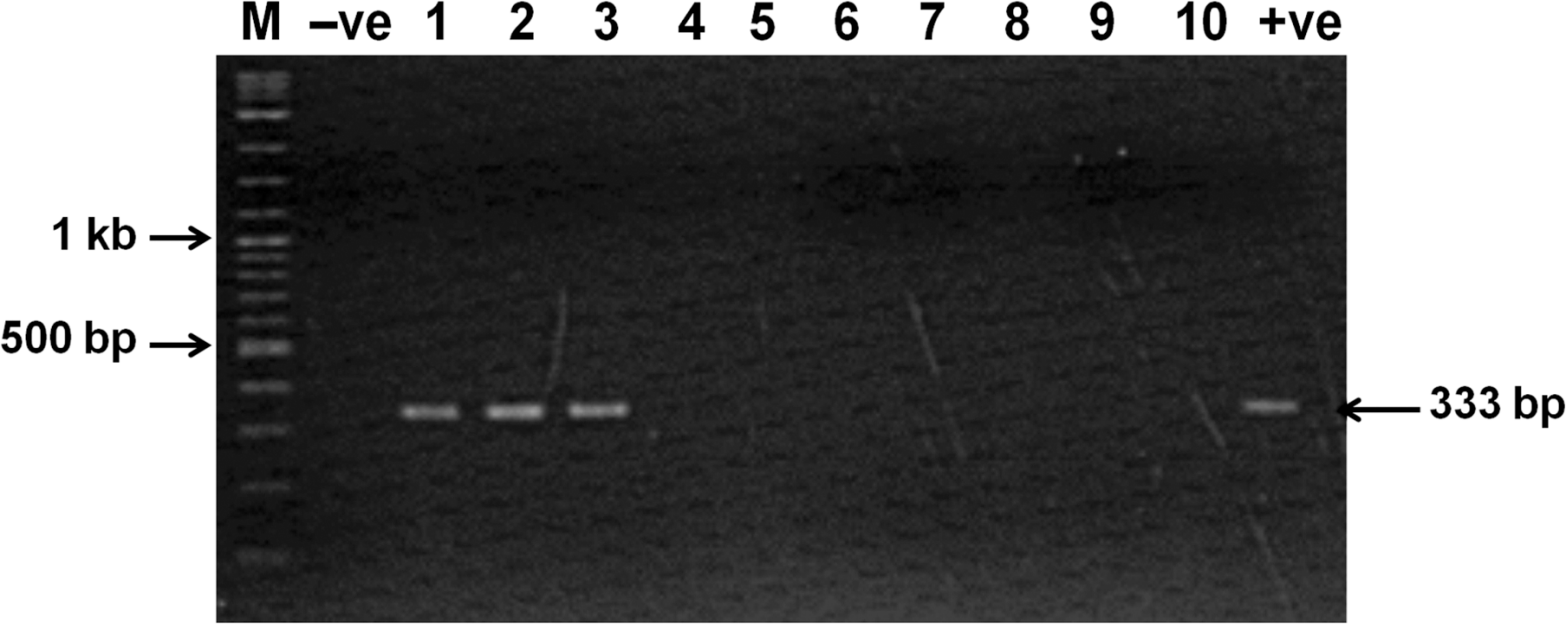
Agarose gel of PCR amplicons from VP_AHPND_ using the AP3 method. Lane M; DNA marker, Lane N: negative control; Lanes 1–3: Positive amplicons (333 bp) from 3 isolates of VP_**AHPND**_ bacteria; Lanes 4–10: No amplicons from 8 non-AHPND bacteria; Lane P: positive control (333 bp).

The materials and methods section described a PCR protocol called the AP3 method for amplification of the complete *ToxA* gene to detect VP_AHPND_ isolates. An example agarose gel is shown in **Fig 6**, showing that VP_AHPND_ isolates (Lanes 1–3) gave positive amplicons with the method (i.e., the presence of an amplicon band at 333bp) while non-AHPND isolates of *V*. *parahaemolyticus* gave no amplicons (Lanes 4–10). To validate the test, a total of 104 bacterial isolates were tested in this way, including 34 non-AHPND VP and 51 VP_AHPND_ isolates (total 85) (all confirmed by Loc Tran bioassay) [1] plus another 19 isolates of bacteria commonly found in shrimp ponds, including other species of *Vibrio* and *Photobacterium* (**Table 2**). Results for all 51 AHPND isolates were positive with the test, while results for all the remaining non-AHPND isolates (i.e., all 53 remaining isolates) were negative. Thus, the AP3 method gave 100% specificity and sensitivity for detection of VP_AHPND_ using the panel of isolates tested. A recent publication using 9 AHPND and 11 non-AHPND isolates of *V*. *parahaemolyticus* from Mexico [3] reported that the AP3 method gave the highest positive (90%) and negative (100%) predictive values out of 5 PCR methods tested.

**Table 2:** Validation of the AP3 method for detection of VP_AHPND_ isolates. The 104 bacterial isolates used were verified as AHPND or non-AHPND isolates by bioassay. Results are also given for detection using the previous interim PCR methods AP1 and AP2. The specimens marked unidentified (*) were not isolates of *Vibrio parahaemolyticus*.

Bacterial isolates	Number of isolates tested	Bioassay result	Number PCR positive		
			AP1	AP2	AP3
*Vibrio alginolyticus*	7	Non-AHPND	0	0	0	
*Vibrio harveyi*	3	Non-AHPND	0	0	0	
*Vibrio vulnificus*	1	Non-AHPND	0	0	0	
*Photobacteriumdamsella*	1	Non-AHPND	0	0	0	
Unidentified*	7	Non-AHPND	0	0	0	
*Vibrio parahaemolyticus*	34	Non-AHPND	3	0	0	
*Vibrio parahaemolyticus*	51	AHPND	48	49	51	
False positive results	53	Non-AHPND	3	1	0	
False negative results	51	AHPND	0	1	0	

As described in the introduction section, two interim PCR detection methods for AHPND bacteria (AP1 and AP2) had been announced at the website of the Network of Aquaculture Centres in Asia-Pacific in December 2013 [5] and subsequent testing revealed that the AP2 method was the best of the two, giving 97% positive predictive value for detection of VP_AHPND_ isolates with a panel of bacterial isolates. Since the target sequences for the AP1 and AP2 methods were initially purported (later confirmed [11]) to be located on the same plasmid as the toxin genes, we hypothesized that the false positive results arose from detection of a virulence plasmid in which the AHPND toxin(s) genes were absent or mutated to inactive form, and that a better detection method would target the toxin gene(s) itself. Results from use of the AP3 method with an extended set of the bacterial isolates used to evaluate AP1 and AP2, gave no false positive or false negative results, while the AP2 method with the same extended set gave 1 false positive result in addition to the 1 false negative result previously reported at the NACA website (Table 2). Thus, the AP3 method was released on 18 June 2014 (also at the NACA website) with the recommendation that the AP3 method replace the interim AP1 and AP2 detection methods.

The AP3 method has been used in Thailand and elsewhere since June 2014, and so far (up to 31 January 2015) the feedback has been positive. Results accumulating from Thailand have revealed that VP_AHPND_ is prevalent in pond water and sediments, in feces of some broodstock, in some batches of post larvae (PL) and in some living animals (or parts thereof) often fed to broodstock shrimp during maturation. This has increased the awareness of the biosecurity risks for VP_AHPND_ facing shrimp PL producers and shrimp farmers and it suggests that application of PCR screening of broodstock and PL will help to decrease the probability of AHPND outbreaks by reducing transmission to rearing ponds via PL. In addition, PCR monitoring during pond preparation and shrimp cultivation may help in identification of environmental reservoirs of VP_AHPND_ so that transmission from them to ponds may be reduced or eliminated.

## Conclusions

Binary toxins called AHPND ToxA (12.7 kDa) and ToxB (50.1kDa) that resemble binary Pir-A&B insecticidal toxins from *Photorhabdus* species in amino acid sequence and in gene arrangement have been isolated and characterized as virulence factors that cause AHPND pathology in shrimp. A specific PCR AP3 detection method for AHPND-inducing bacteria was designed based on the ToxA gene and it is recommended that the method be used to monitor shrimp broodstock, to screen living or raw, unfrozen animals or animal parts fed to broodstock and to test post-larvae before stocking ponds. Positive shrimp or feeds should be suitably destroyed to prevent dispersal of the target pathogen.
